# Most oxytocin administration studies are statistically underpowered to reliably detect (or reject) a wide range of effect sizes

**DOI:** 10.1016/j.cpnec.2020.100014

**Published:** 2020-10-26

**Authors:** Daniel S. Quintana

**Affiliations:** a NORMENT, Division of Mental Health and Addiction, University of Oslo, And Oslo University Hospital, Oslo, Norway; b Department of Psychology, University of Oslo, Oslo, Norway; c KG Jebsen Centre for Neurodevelopmental Disorders, University of Oslo, Norway

**Keywords:** Oxytocin, Social behaviour, Neuroendocrinology, Statistics

## Abstract

The neuropeptide oxytocin has attracted substantial research interest for its role in behaviour and cognition; however, the evidence for its effects have been mixed. Meta-analysis is viewed as the gold-standard for synthesizing evidence, but the evidential value of a meta-analysis is dependent on the evidential value of the studies it synthesizes, and the analytical approaches used to derive conclusions. To assess the evidential value of oxytocin administration meta-analyses, this study calculated the statistical power of 107 studies from 35 meta-analyses and assessed the statistical equivalence of reported results. The mean statistical power across all studies was 12.2% and there has been no noticeable improvement in power over an eight-year period. None of the 26 non-significant meta-analyses were statistically equivalent, assuming a smallest effect size of interest of 0.1. Altogether, most oxytocin treatment study designs are statistically underpowered to either detect or reject a wide range of worthwhile effect sizes.

## Highlights

• The mean statistical power across 107 oxytocin administration studies was 12.2%.
• The was no noticeable improvement in statistical power over an eight-year period.
• None of the 26 non-significant meta-analyses were statistically equivalent.
• Most oxytocin studies cannot detect or reject a wide range of effect sizes.

## 1 Introduction

Oxytocin is an evolutionarily ancient neuromodulator that is mainly synthesized in the hypothalamus and released both centrally and peripherally to exert effects on several organ systems [1]. While the effects of oxytocin on childbirth and lactation are well-established, oxytocin has more recently attracted immense research interest for its role in both social and non-social cognition and behaviour [2]. Preliminary results from animal and human research led to the tantalizing proposal that oxytocin administration may help ameliorate social impairments in various psychiatric illnesses, such as autism [3]. But despite this early promise, subsequent studies investigating the effects of oxytocin administration on cognition and behaviour have generated mixed results and some initial findings (e.g., oxytocin increases trusting behaviors) have failed to replicate [4].

Several meta-analyses on the effects of oxytocin administration have been conducted to better understand these mixed results. Although meta-analysis is widely seen as the gold-standard for evidence sythesis, the evidential value of a meta-analysis and its conclusions are seldom evaluated. A non-significant result is typically associated with the absence of an effect. However, when using a traditional null-hypothesis significance test alone it is impossible to tease apart whether a non-significant result is due to an insensitive design or the absence of an effect. While it is practically impossible to identify an effect size is exactly zero, equivalence testing can be used to reject *a range* of effect sizes that are theoretically or practically interesting [5]. For example, if a standardized mean difference (δ) greater than or equal to 0.1 is considered worthwhile, then an equivalence test can reject the presence of these worthwhile effects. A non-significant equivalence test in this case would suggest that the design was not sensitive enough to reject effect sizes of δ ​≥ ​0.1. Like traditional null hypothesis significance tests, equivalence tests require appropriate statistical power to reliably reject effect sizes of interest. While equivalence tests are typically applied to statistical tests from individual datasets, they can also be applied to meta-analytic summary effect sizes, which are estimated via the synthesis of multiple datasets.

Recent work suggests that non-significant results reported from oxytocin administration studies tend to be derived from insensitive research designs [6,7]. That is, while results from many of these studies were not statistically significant, they were not statistically equivalent either. While this research illustrates the importance of more closely examining non-significant results, these studies were conducted on a limited sample of intranasal oxytocin studies. Research has yet to examine statistical equivalence of the larger body of oxytocin administration studies and whether research designs have been improving over time to become more sensitive to detect or reject a wider range of effect sizes. Therefore, the present study performed equivalence tests on published meta-analyses and calculated the statistical power of intranasal oxytocin administration studies included in these studies to determine the range of effects that can be reliably detected or rejected.

## 2 Materials and methods

Meta-analyses that assessed the impact of oxytocin administration on cognition and behaviour using frequentist statistics were extracted from PubMed and Web of Science on June 14, 2020 using the following search string: (oxytocin[Title/Abstract]) AND (*meta*-analy∗[Title/Abstract]). Effect size measures of standardized mean differences (δ), which included Hedges’ *g*, Cohen’s *d*, and standardized mean change values, and standard errors were extracted from eligible studies. If standard errors were not available, these were calculated using confidence intervals. Data from meta-analyses that used negative values to represent a positive outcome after oxytocin administration (n ​= ​8) were reversed, so that all effects were in the same direction.

Equivalence tests on the summary effect size estimates were calculated using the ‘TOSTER’ R package [5] using three different equivalence bounds: δ ​= ​0.1, δ ​= ​0.2, and δ ​= ​0.3. These bounds were selected in light of the small meta-analytic effects generally reported in the oxytocin treatment literature. The statistical power for each study was calculated using an adapted function from the ‘metaviz’ R package [8], which calculates the statistical power associated with a given standard error via a two-sided Wald test. As some studies were included in multiple meta-analyses, a separate analysis that only included effect sizes from unique studies was performed. The median power was used if multiple tests were reported from the same study. The Test of Excess Significance [9] was used to examine whether there was a higher than expected number of statistically significant studies in the 18 univariate meta-analyses. The datafile, generated datasets, and R analysis scripts to reproduce the analyses and figures are available at https://osf.io/86jvm/.

## 3 Results

**Fig. 1:**
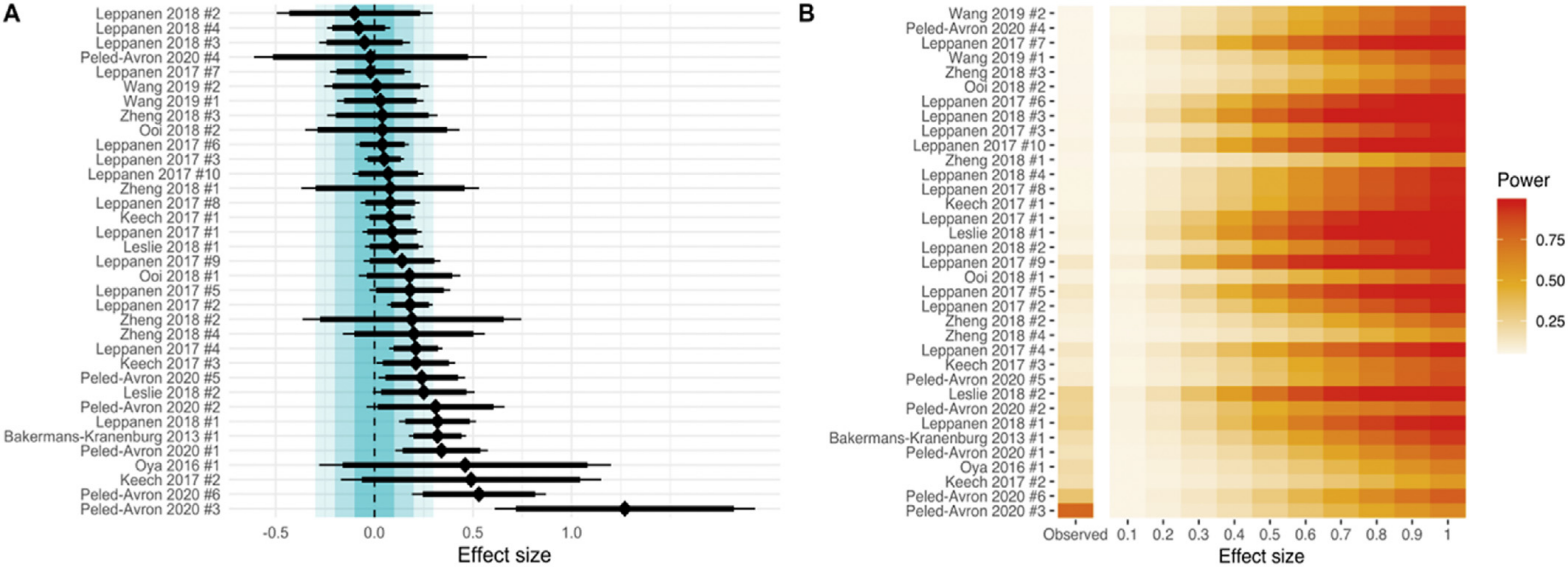
Effect sizes (diamonds) with 95% null hypothesis significance test confidence intervals (thin lines) and 90% two one-sided test (TOST) confidence intervals (thick lines) for 35 oxytocin administration meta-analyses are shown in panel A. The dark blue zone represents a 0.1 equivalence bound, the mid-blue zone a 0.2 equivalence bound, and the light blue zone represents a 0.3 equivalence bound. No TOST confidence interval fell within the 0.1 equivalence bound suggesting that no meta-analysis summary effect size estimate is statistically equivalent at this level. In panel B, the median power for studies included in each meta-analysis is shown, assuming a range of true effect sizes. Statistical power using the observed summary effect size estimate as the true effect size estimate is also shown. (For interpretation of the references to colour in this figure legend, the reader is referred to the Web version of this article.)

The database search returned 255 potentially eligible meta-analysis articles. Ten meta-analysis articles were eligible for inclusion (Supplementary Fig. 1), containing 35 meta-analyses with 107 unique articles (for full details of each meta-analysis and studies included in the analysis, see https://osf.io/86jvm). The median summary effect size for standardized mean differences across the 35 meta-analyses was 0.14 (mean ​= ​0.18, min ​= ​−0.1, max ​= ​1.27). Out of these 35 meta-analyses, 9 reported a statistically significant effect, in which oxytocin administration was beneficial for the outcome of interest (Fig. 1A). Of the remaining 26 meta-analyses that did not report a statistically significant effect, none demonstrated a statistically equivalent result, assuming a smallest effect size of interest (SESOI) of δ ​= ​0.1. In other words, no meta-analysis was sensitive enough to reject effects of δ ​≥ ​0.1. When assuming a larger SESOI of δ ​= ​0.2, 4 out of 26 non-significant studies (15.4%) demonstrated statistical equivalence. With a SESOI of δ ​= ​0.3, 13 out of 26 non-significant studies (50%) demonstrated statistical equivalence.

**Fig. 2:**
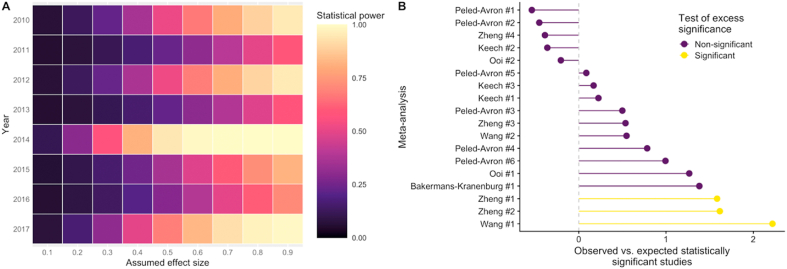
The statistical power of oxytocin administration studies from 2010 to 2017 is presented for a range of assumed true effect sizes (δ) (A). Three out of eighteen univariate meta-analysees had a higher than expected number of statistically significant studies than expected (B).

The median statistical power of the studies included in each meta-analysis, using the observed summary effect size estimate as the true effect size, was 8.1% (mean ​= ​12.8%, min ​= ​5%, max ​= ​76.8%). The median statistical power of the studies included in each meta-analysis for a range of true effect sizes is presented in Fig. 1B, which indicates that for most areas of oxytocin administration research, studies are generally designed to only detect effect sizes that are conventionally categorised as medium-to-large. When only using effect sizes from unique studies (n ​= ​107), there was a median statistical power of 7.8% (mean ​= ​12.2%, min ​= ​5%, max ​= ​97.7%). There were 68 unique studies in clinical populations (mean statistical power ​= ​12.1%; SD ​= ​13.5%) and 39 unique studies in healthy populations (mean statistical power ​= ​12.3%; SD ​= ​10.7%), but there was no significant difference in statistical power between these study categories [*t*(94.6) ​= ​−0.06, *p* ​= ​0.096]. This effect was not statistically equivalent when using a SESOI of δ ​= ​0.2 [*t*(94.6) ​= ​0.97, *p* ​= ​0.16]. There were 30 unique studies in clinical populations observing the effects of multiple oxytocin doses (mean statistical power ​= ​14.6%; SD ​= ​18.9%) and 38 unique studies in clinical populations evaluating the effects of a single oxytocin dose (mean statistical power ​= ​10.3%; SD ​= ​6.4%), but there was no significant difference in statistical power between these study categories [*t*(34.3) ​= ​1.18, *p* ​= ​0.25]. These effects were not statistically equivalent when using a SESOI of δ ​= ​0.2 [*t*(34.3) ​= ​0.4, *p* ​= ​0.65]. The year-to-year statistical power (2010–2017, n ​= ​104) for a range of true effect sizes is illustrated in Fig. 2A. A one-way ANOVA indicated no significant change over time between in statistical power for individual studies [F(14,89) ​= ​0.89, *p* ​= ​0.57].

Publication bias, in which non-significant results are less likely to be published, is an ongoing issue for meta-analysis. Meta-analyses typically use Egger’s regression tests to address this problem, but this tools assesses several sources of small-study bias beyond publication bias [10]. Using the Test of Excess Significance [9] to specifically assess publication bias for the 18 univariate meta-analyses revealed that three had more statistically significant results than expected, given the data (p ​< ​0.05; Fig. 2B).

## 4 Discussion

Most oxytocin administration studies are unable to reliably detect or reject a wide range of meaningful effect sizes. Despite repeated calls to increase statistical power (e.g. Ref. [11]), there has not been any considerable change over an eight-year period. While the effects of underpowered studies are often framed in terms of their influence on significant results there is comparatively little attention given towards the effects of underpowered studies on results that are not statistically significant, which can thwart hypothesis falsification. In the current sample, no meta-analysis outcome was statistically equivalent at a δ ​= ​0.1 level. When using a level of δ ​= ​0.2 for statistical equivalence, 4 out of 26 non-significant meta-analyses demonstrated statistical equivalence. While this a noteworthy result, the assumption for this test is that effect sizes less than δ ​= ​0.2 are not worthwhile. Considering that the median effect size across these meta-analyses was 0.14, and that it is likely that these effects are inflated due to publication bias [12], specifying effects less than δ ​= ​0.2 as “not worthwhile” is probably too conservative. However, for the 18 univariate meta-analyses included in the overall analysis, only 3 included a higher than expected number of statistically significant studies, suggesting that publication bias does not appear to be a widespread issue in the field, at least for primary analyses reported in papers. When assuming that effects less than δ ​= ​0.3 are not worthwhile, which might be relevant for some resource-intensive clinical interventions, half of the non-significant studies (13 out of 26) demonstrated statistical equivalence. To evaluate statistical equivalence using other SESOIs, interested readers can access the summary data and analysis scripts online https://osf.io/86jvm.

Underpowered research designs that cannot detect or reject a wide range of effects are usually a result of resource constraints, such as a lack of finances or time. One approach for operating under such conditions is to be explicit about the effect sizes that can be reliably detected. If a study design can only reliably detect an effect size of δ ​≥ ​0.5, for instance, then the study architects would need to accept that this is a plausible effect for a given intervention and be satisfied with the fact that effect sizes of δ ​< ​0.5 that cannot be reliably detected or rejected. This trade-off might be worthwhile in some cases, such as the study of rare disease populations in which recruitment of large samples is unrealistic. While a single lab may not have the capability of recruiting large samples size, labs can pool resources across multiple sites (if possible) so that a wider range of effect sizes can be reliably be detected or rejected (e.g., Ref. [4]).

The present analysis synthesized studies across several research areas, so it is possible that there are differences in study design between sub-fields of oxytocin administration research that influence statistical power. Indeed, there are individual examples of sufficiently powered research studies that were included (e.g. Ref. [13]), and not included (e.g. Refs. [4,14]), in the present analysis, therefore the main conclusion is not applicable to *all* oxytocin administration studies. In terms of potential moderator effects, there was no significant difference in statistical power between studies that recruited healthy populations and clinical populations, nor studies that evaluated a single oxytocin administration compared to multiple administrations. A limitation of the present research design is that there is a considerable time-lag between study planning and publication, so it is possible that improved research practices are yet to be reflected in the published research record. The archived and publicly available data and scripts from the present analysis will facilitate future re-evaluation of this research question, as new data can be added when available and analyses re-ran using the open script.

Ultimately, these results highlight how study design can influence the range of effect sizes that can be reliably detected or rejected in oxytocin administration research. It is possible that the effects of oxytocin administration are small, yet clinically interesting. However, current research designs are not typicallyty equipped to determine this. Conversely, the data from the present study indicates that in general, study designs are also poorly equipped to reject effects that as large (or larger) than δ ​= ​0.1. Whether explicit or not, researchers make a statement about the range of effect sizes that they not interested (or capable) of reliably detecting when designing a study. For oxytocin administration research to date, this range of effect sizes that cannot be reliably detected is relatively wide. The utility of oxytocin administration for the treatment of psychiatric illness and the realisation that particular research lines need to be abandoned or adapted will be more rapidly recognised by designing studies that are sensitive enough to detect and reject a wide range of effect sizes.

## Declaration of competing interest

The author declares that they have no known competing financial interests or personal relationships that could have appeared to influence the work reported in this paper.
