# Supplementary figures and images for: Most oxytocin administration studies are statistically underpowered to reliably detect (or reject) a wide range of effect sizes
Source: Compr Psychoneuroendocrinol. 2020 Oct 26;4:100014. doi: 10.1016/j.cpnec.2020.100014 (PMC9216440; doi:10.1016/j.cpnec.2020.100014)

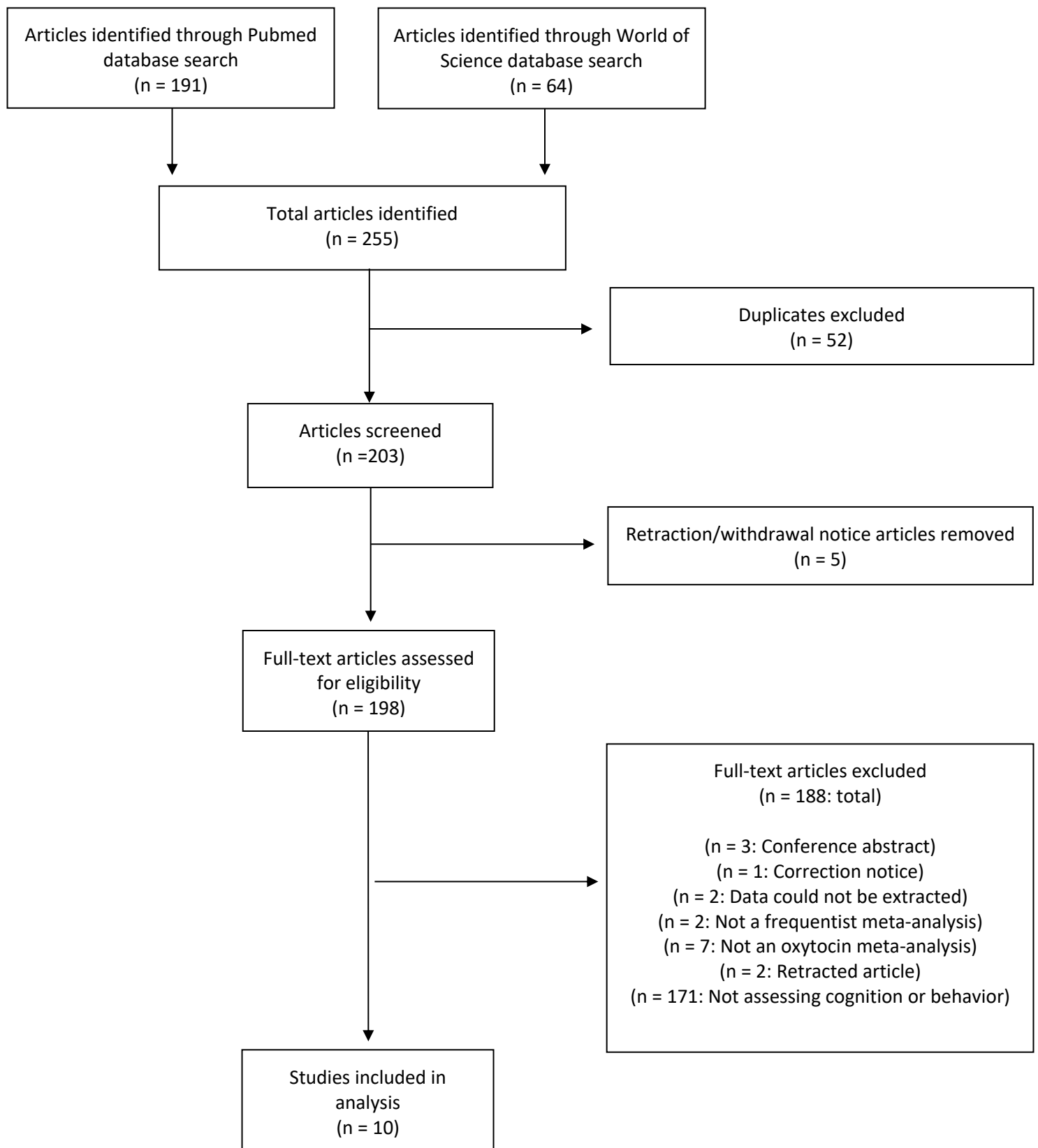

**Supplementary Figure 1: Flow diagram of meta-analysis selection.**

Supplement: Multimedia component 1 [file mmc1.pdf]
